# Effect of Amorphous Metallic Fibers on Thermal and Mechanical Properties of Lightweight Aggregate Cement Mortars Containing Carbon Nanotubes

**DOI:** 10.3390/ma17225449

**Published:** 2024-11-08

**Authors:** Se-Jin Choi, Jae-In Lee, Chae-Young Kim, Joo-Ho Yoon, Kwan-Ho Kim

**Affiliations:** Department of Architectural Engineering, Wonkwang University, 460 Iksan-daero, Iksan 54538, Republic of Korea; csj2378@wku.ac.kr (S.-J.C.); cykim043@naver.com (C.-Y.K.); yoonjh0091@naver.com (J.-H.Y.); kwanho97@naver.com (K.-H.K.)

**Keywords:** artificial lightweight aggregate, carbon nanotube, amorphous metallic fiber, thermal property, compressive strength

## Abstract

Lightweight aggregate concrete can reduce the self-weight of a structure with a low unit weight; however, disadvantages such as reduced strength and brittleness remain. This study evaluated the thermal and mechanical properties of lightweight aggregate cement mortars containing carbon nanotubes (CNTs) and amorphous metallic fibers (AMFs). A thermal property test indicated that the peak temperature of the C1A1 and C1A2 samples using AMFs was approximately 91.5–93.8 °C (approximately 57.2–61.1% higher than the C1A0 sample without AMFs). The time to reach the peak temperature was approximately 15–27 min (21.1–38.0% of that for the C1A0 sample). The 28-day split tensile strength of the sample using 20 kg/m^3^ of the AMFs was approximately 3.6–3.8 MPa (approximately 46.1–50.0% higher than that of CNT-only samples). The 56-day flexural strength of the C2A2 sample using 0.2% CNTs and 20 kg/m^3^ AMFs was the highest at approximately 11.2 MPa (approximately 24.4% higher than that of the control sample). The results of this study indicate that using CNTs and AMFs can enhance the strength and reduce the brittleness of lightweight aggregate cement mortar. Furthermore, the performance of the cement mortar is significantly improved when combined with AMFs compared to using CNTs alone.

## 1. Introduction

Lightweight aggregate (LWA) concrete is a building material used in high-rise buildings and marine floating structures [1,2] owing to its low unit weight and dead load reduction effect [3,4]. However, there are certain disadvantages, such as the reduced performance of the concrete due to the porosity of artificial LWAs [5], low strength, and high brittleness [6,7].

To compensate for these shortcomings, certain studies have reported improvements in the performance of LWA concrete using fibers with high tensile strength and ductility enhancement effects as reinforcing materials [8,9]. Amorphous metallic fibers (AMFs), which are concrete-reinforcing fibers, exhibit high adhesion to the cement matrix, excellent tensile strength, and corrosion resistance. Moreover, they are known to improve mechanical properties and durability when incorporated into cement composites [10,11].

Dang et al. [8] evaluated the mechanical performance of a lightweight mortar using amorphous metals and nylon fibers. Consequently, they reported that the bending strength increased with an increase in the proportion of AMFs. Moreover, the length of nylon fibers exerted a significant effect on the deformation ability at maximum load. A study by Choi et al. [9] examined the strength and durability of AMF-reinforced mortar using artificial LWA. The bending strength increased with an increase in the mixing ratio of AMFs. Choi et al. [10] reviewed the corrosion resistance and plastic shrinkage characteristics of AMFs. They reported that AMFs exhibited higher corrosion resistance and crack control performance than general steel fibers in deteriorating environments. Zhao et al. [11] evaluated the performance of cement composites containing 0.4, 0.8, 1.2, and 1.6% AMFs. It was found that mixing more than 1.2% of AMFs had a negative effect on the chloride ion penetration resistance.

Recently, it has been reported that the performance of LWA concrete can be improved by incorporating nanomaterials, such as carbon nanotubes (CNTs) and graphene [12,13,14]. Carbon exhibits high efficiency even in small amounts [15]. Furthermore, it exhibits extremely high conductivity, and when incorporated into cement composites, it improves the conductivity of cement composites by forming a conductive network [16].

Adhikary et al. [12] reviewed the effects of CNTs and graphene nanoplates on lightweight concrete. They reported that the compressive strength of the sample using CNTs was higher than that of the sample using graphene nanoplates. Further, the compressive strength of the sample combining CNTs and graphene nanoplates was reported to be the highest. Du et al. [13] evaluated the properties of a lightweight mortar containing CNTs and nanosilica. It was found that the incorporation of nanomaterials improved the mechanical properties of lightweight mortar and that the porosity decreased with the incorporation of approximately 0.05–0.15% CNTs. Yoo et al. [14] evaluated the electrical resistance of cement paste using CNTs and graphene and reported that the maximum decrease in the electrical resistance was observed in the sample that used approximately 1% CNTs.

However, studies on LWA cement composites using CNTs with high conductivity and AMFs with excellent tensile strength and corrosion resistance are lacking.

Thus, this study evaluated the thermal and mechanical properties of LWA cement mortars containing CNTs and AMFs. The fluidity, microhydration heat, strength, thermal properties, and microstructure of LWA mortars containing 0, 0.1, and 0.2% CNTs and 0, 10, and 20 kg/m^3^ AMFs were examined.

## 2. Materials and Experimental Methods

### 2.1. Materials

The cement used in this study was ordinary Portland cement (Sampyo Cement, Seoul, Republic of Korea) with a specific gravity of 3.13 g/cm^3^ and a Blaine fineness of 3820 cm^2^/g. For the fine aggregate, artificial lightweight fine aggregate (Koen, Jinju, Republic of Korea) with a specific gravity of 1.77 g/cm^3^ and a fineness modulus of 4.61, manufactured by firing coal ash and dredged soil at 1200 °C, was used. In addition, we used multi-walled CNTs and AMFs (SEVA, Chalon-sur-saône, France) as reinforcing materials.

Table 1 and Table 2 list the chemical properties of cement and the physical properties of the artificial LWA used in this study, respectively. Table 3 and Table 4 present the characteristics of CNTs and AMFs used in this study, respectively.

**Figure 1:**
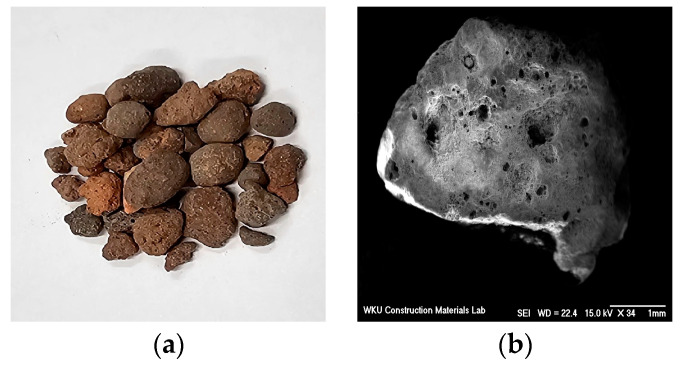
Lightweight fine aggregate: (**a**) shape, and (**b**) SEM image.

**Figure 2:**
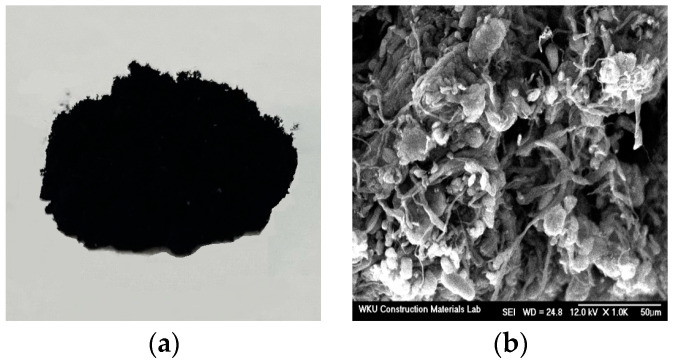
Carbon nanotube: (**a**) appearance, and (**b**) SEM image.

**Figure 3:**
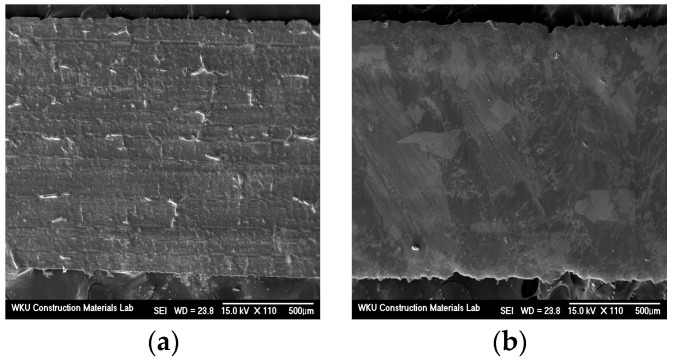
SEM images of AMF: (**a**) rough surface, and (**b**) smooth surface.

Figure 1 shows the shape and scanning electron microscopy (SEM) images of the LWA. Evidently, the LWA was porous and had a high water absorption rate, which can affect the performance of the mortar. Accordingly, on the basis of a prior study [17], we immersed the LWA in water for 24 h and then adjusted it to a saturated-surface-dry state. Figure 2 and Figure 3 show SEM images of the CNT and AMF used in this study, respectively. It is evident that the surface of the AMF was rough and smooth.

### 2.2. Mixing Proportions and Specimen Preparation

Table 5 lists the mix proportions of the LWA mortars used in this study. The water–cement ratio (W/C) was fixed at 50%, and LWA was used as the fine aggregate. The CNT was added in amounts of 0, 0.1, and 0.2% by cement content, and the AMF was added in amounts of 0, 10, and 20 kg/m^3^. To disperse the hydrophobic CNTs, they were first incorporated in the dry mixing process of the cement and aggregates. Then, the AMF was added to the mortar dough mixed with water.

A 50 mm × 50 mm × 50 mm cubic sample was produced to evaluate the compressive strength and thermal performance of LWA mortar using CNTs and AMFs, and a Ø50 × 100 mm cylindrical sample was produced for the split tensile strength test. Further, a 40 mm × 40 mm × 160 mm prismatic sample was manufactured, and its flexural strength was measured. Microstructural analyses were performed through SEM and energy-dispersive X-ray spectroscopy (EDS). Each sample was demolded after 24 h and cured in water at 20 °C until further use.

**Figure 4:**
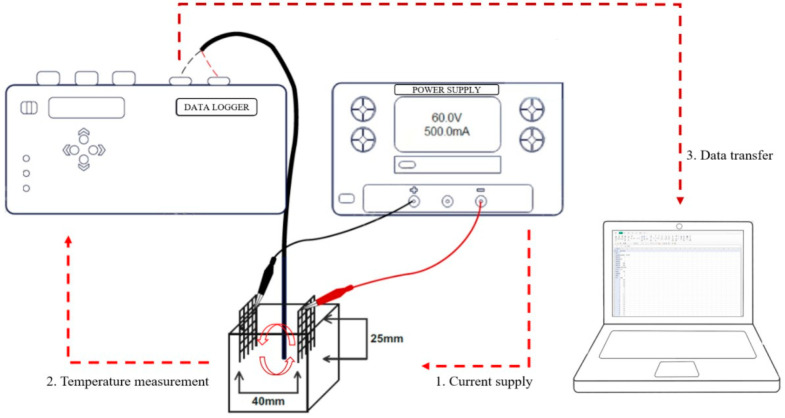
Thermal test setup.

The mortar flow and compressive strength were measured according to KS L 5105 [18]. The heat of microhydration was measured according to ASTM C 1753 [19] using a semiadiabatic calorimeter (Calmetrix, F-Cal8000, Boston, MA, USA). For the thermal characteristics, referred to in the existing literature [20,21], a K-TYPE thermocouple was used. Temperature monitoring was conducted until the maximum temperature was reached after applying 60 V (Figure 4). For sample preparation, a cubic test specimen of 50 mm × 50 mm × 50 mm was created, and a thermocouple was inserted into the center of the test specimen at a depth of 25 mm before the material hardened, with electrode bodies inserted at intervals of 40 mm. The samples were cured in water at 20 °C prior to thermal performance measurement, having been cured at room temperature for 24 h before measurement to minimize the effect of moisture . The split tensile strength was measured according to KS F 2423 [22], and the flexural strength was tested according to KS F 2408 [23]. The microstructures were analyzed via SEM (AIS1800C, Seron, Uiwang-si, Republic of Korea) and EDS (OXFORD INSTRUMENTS, Xplore, Abingdon, UK).

## 3. Experimental Results and Discussion

### 3.1. Mortar Flow

**Figure 5:**
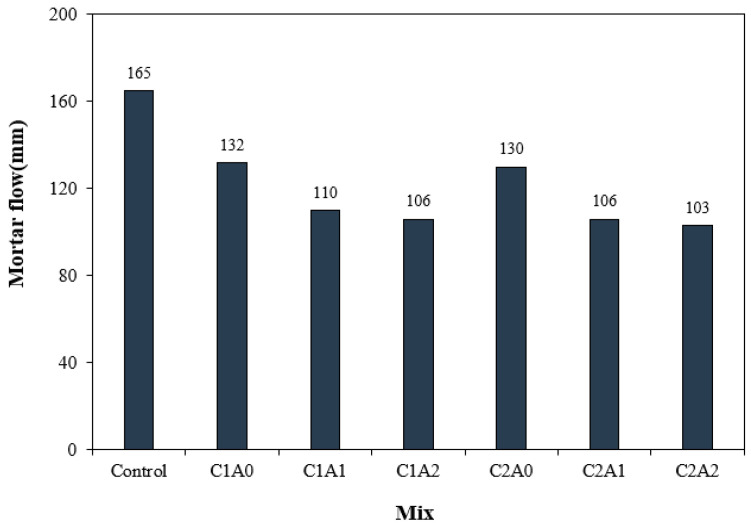
Mortar flow test results.

Figure 5 shows the flow change in LWA mortar using CNTs and AMFs. Evidently, the mortar flow of the control sample without CNTs and AMFs was approximately 165 mm, which was the highest among all the samples. The mortar flows of the C1A0 and C2A0 samples using CNTs without AMFs were approximately 130–132 mm, approximately 20.0–21.2% lower than that of the control sample. When a CNT was used, the mortar flow of the C1A0 sample using 0.1% CNTs and 0 kg/m^3^ AMFs was the highest at approximately 132 mm.

In addition, as the amount of AMFs increased, the flow of the mortar sample decreased, and the flows of the C1A1 and C1A2 samples were approximately 16.6–19.6% lower than that of the C1A0 sample without AMFs. Even in the sample with 0.2% CNTs, the mortar flow decreased as the amount of AMFs increased, and the mortar flow of the C2A2 sample containing 20 kg/m^3^ AMFs was the lowest at approximately 103 mm.

The decrease in the mortar flow when using CNTs is attributed to an increase in the agglomeration rate of the hydrophobic CNTs [24]. Additionally, the tendency of mortar flowability to decrease when using AMFs is believed to result from the uneven distribution of fibers as the amount of AMFs increases [25] and moisture loss, which can affect fluidity when dry fibers are mixed in. When CNTs and AMFs were used together, the mortar flowability was found to decrease further, aligning with findings from a previous study that reported reduced fluidity when CNTs and reinforcing fibers were combined [26].

### 3.2. Heat of Microhydration

**Figure 6:**
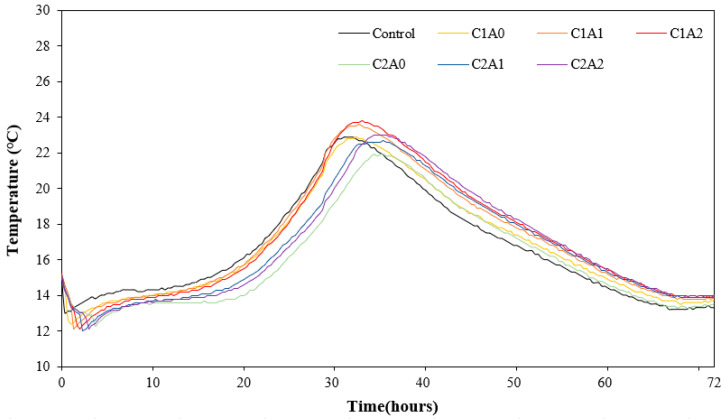
Heat of microhydration test results.

Figure 6 shows the change in the microhydration heat of the LWA mortar samples using CNTs and AMFs. Evidently, the control sample showed the fastest time to reach peak temperature, that is, 22.9 °C in approximately 30 h. In the case of the sample containing 0.1% CNTs, the peak temperature reaching time was approximately 32–34 h, which was slightly delayed compared with the control sample.

Further, as the amount of AMFs increased, the peak temperature increased to 23.5–23.8 °C, which was approximately 2.6–3.9% higher than that of the C1A0 sample. In the case of the sample using 0.2% CNTs, the peak temperature of the C2A0 sample without AMFs was approximately 21.9 °C, which was approximately 4.3% lower than the control sample. Thereafter, as the amount of AMFs increased, the peak temperature tended to increase to 22.6–23.0 °C.

The results of this study show that when only a CNT is used in LWA mortar, the time to reach the peak temperature is delayed. CNT agglomerates can potentially delay the hydration reaction by reducing the homogeneity of the cement matrix and the contact interface between the pastes [27,28]. However, when CNTs and AMFs are used together, the peak temperature increases. This trend is attributable to the agglomeration phenomenon of CNTs [29,30] being partially alleviated using the AMF, which acts as a medium for transferring hydration heat.

### 3.3. Thermal Properties

**Figure 7:**
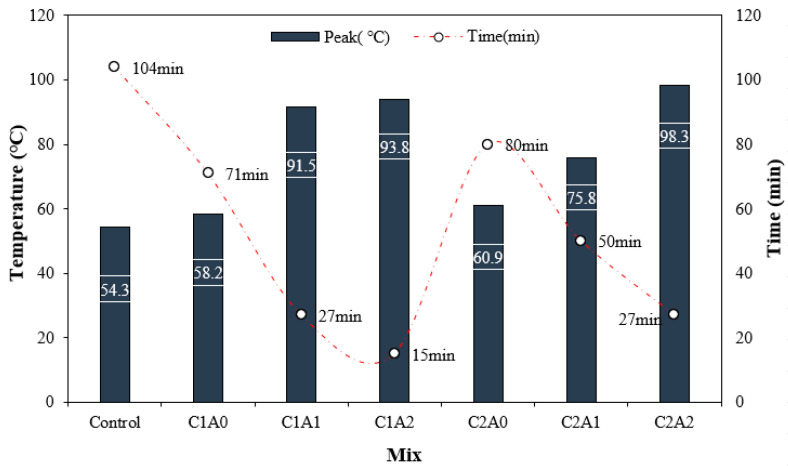
Peak temperature and reaching time.

Figure 7 shows the changes in the thermal properties of the LWA mortar using CNTs and AMFs. The peak temperature and arrival time of the control sample were approximately 54.3 °C and 104 min, respectively, which were the lowest and slowest, respectively.

The peak temperature of the C1A0 and C2A0 samples containing only CNTs was approximately 58.2–60.9 °C, approximately 7.1–12.1% higher than the control sample. Further, the peak temperature of the sample containing both CNTs and AMFs was significantly higher than that of the sample containing only CNTs.

Thus, in the case of the sample using 0.1% CNTs, the peak temperature of the LWA mortar sample using AMFs was approximately 91.5–93.8 °C, which was approximately 57.2–61.1% higher than the C1A0 sample without AMFs.

In particular, the time to reach the peak temperature was approximately 15–27 min, which was 21.1–38.0% of that of the C1A0 sample. Even when 0.2% of the CNT was used, the peak temperature of the sample using AMFs was approximately 75.8–98.3 °C, which was approximately 24.4–61.4% higher than that of the C2A0 sample. Moreover, the time required to reach the peak temperature was also short. The peak temperature of the C2A2 sample using 0.2% CNTs and 20 kg/m^3^ AMFs was the highest at approximately 98.3 °C, which was approximately 81.0% higher than the control sample.

In this study, the thermal performance of LWA mortar was significantly improved when CNTs and AMFs were used together as reinforcing materials. This is attributed to the formation of a conductive network in the mortar sample owing to the incorporation of CNTs [31] and the significant increase in thermal performance owing to the use of conductive AMFs [32].

### 3.4. Compressive Strength

**Figure 8:**
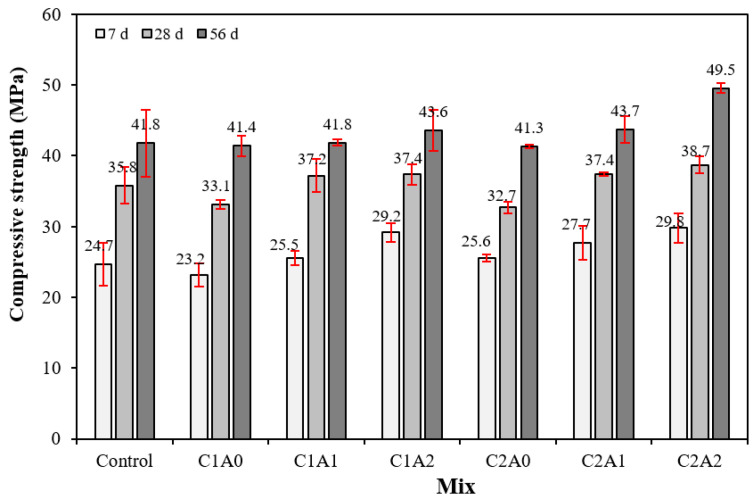
Compressive strength test results.

Figure 8 shows the change in compressive strength with the age of the LWA mortar samples using CNTs and AMFs. Notably, the 7-day compressive strength of the control sample was approximately 24.7 MPa, and as the amount of CNTs and AMFs increased, the compressive strength of the LWA mortar sample increased. In addition, when the amount of AMFs was the same, the compressive strength of the sample with 0.2% CNTs was greater than that of the sample with 0.1% CNTs.

The 7-day compressive strength of the C2A2 sample using 0.2% CNTs and 20 kg/m^3^ AMFs was approximately 29.8 MPa, which was approximately 20.6% higher than that of the control sample.

After 28 days, the compressive strength of the control sample was approximately 35.8 MPa, and the 28-day compressive strength of the samples using CNTs and AMFs increased as the amount of AMFs increased. The improvement in the compressive strength of all samples continued even after 56 days. The 56-day compressive strength of the control sample was approximately 41.8 MPa, and that of the sample using CNTs and AMFs was 41.3–49.5 MPa, demonstrating an increase with the amount of CNTs and AMFs. The 56-day compressive strength of the C2A2 sample using 0.2% CNTs and 20 kg/m^3^ AMFs was approximately 49.5 MPa, which was approximately 18.4% higher than that of the control sample.

The compressive strength of the LWA mortar using CNTs and AMFs being higher than that of the control sample is attributable to the crosslinking effect of CNTs, a nanomaterial, and the improved adhesion strength to the cement matrix due to the rough surface characteristics of AMFs [33,34,35].

### 3.5. Split Tensile Strength

**Figure 9:**
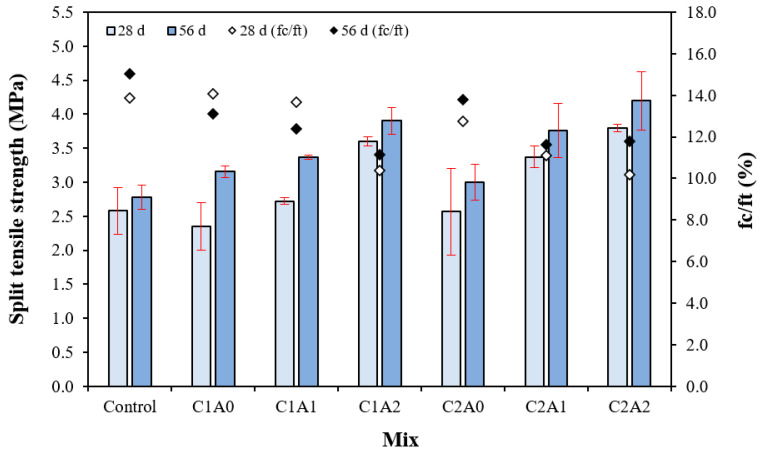
Split tensile strength and fc/ft.

Figure 9 shows the change in the split tensile strength of the LWA mortar samples using CNTs and AMFs. fc/ft is the ratio of the split tensile strength (ft) to compressive strength (fc). The 28-day split tensile strength of the control sample was approximately 2.6 MPa, and for the sample using CNTs and AMFs, it was approximately 2.4–3.8 MPa. The split tensile strength increased with the amount of AMFs. The 28-day split tensile strengths of the C1A0 and C2A0 samples using only CNTs were approximately 2.4–2.6 MPa, similar to that of the control sample.

However, the 28-day split tensile strength of the sample using 10 kg/m^3^ AMFs was approximately 2.7–3.4 MPa, which was approximately 12.5–30.7% higher than that of the sample using only CNTs. In addition, the 28-day split tensile strength of the sample using 20 kg/m^3^ of AMFs was approximately 3.6–3.8 MPa, which was approximately 46.1–50.0% higher than that of the sample using only CNTs.

In particular, when the amount of AMFs was the same, the split tensile strength of the sample with 0.2% CNTs was greater than that of the sample with 0.1% CNTs.

Even after 56 days, the split tensile strength of the control sample was approximately 2.8 MPa, whereas that of the samples using CNTs and AMFs was relatively high at approximately 3.0–4.2 MPa. The 56-day split tensile strength of the C1A2 and C2A2 samples using 20 kg/m^3^ of AMFs was approximately 3.9–4.2 MPa, which was 39.2–50.0% greater than that of the control sample.

The split tensile strengths of the samples prepared using CNTs and AMFs were significantly greater than that of the control sample. This is believed to be owing to the CNT-suppressing microcracks in the mortar [36,37] and the stress distribution effect of the AMFs used as reinforcing fibers [38].

In addition, the brittleness of cement composites is high when the fc/ft is high [32,39]. After 28 days, the fc/ft of the control sample was approximately 13.9, and that of the sample using CNTs and AMFs together was 10.2–13.7—up to 26.6% lower than that of the control sample. Therefore, it is believed that the brittleness of LWA mortar samples can be effectively reduced using CNTs and AMFs.

### 3.6. Flexural Strength

**Figure 10:**
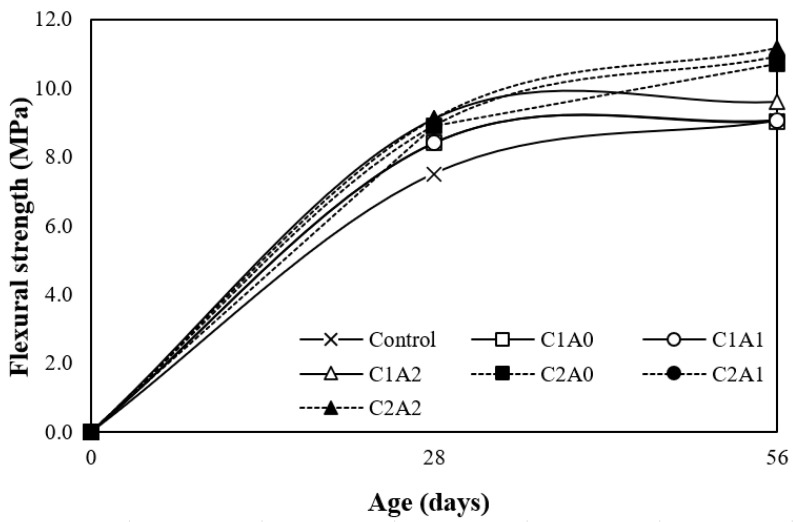
Flexural strength test results.

Figure 10 shows the change in the flexural strength according to the age of the LWA mortar samples using CNTs and AMFs. Evidently, the 28-day flexural strength of the control sample was the lowest at approximately 7.5 MPa.

For the sample with 0.1% CNTs, the flexural strength of the sample without AMFs was approximately 8.4 MPa, and the flexural strength tended to increase as the amount of AMFs increased. The 28-day flexural strength of the sample containing 0.1% CNTs and 20 kg/m^3^ AMFs was approximately 9.1 MPa, which was approximately 21.3% higher than that of the control sample. Even when 0.2% of the CNT was used, the flexural strength increased as the amount of AMFs increased, and the 28-day flexural strength of the C2A2 sample containing 0.2% CNTs and 20 kg/m^3^ AMFs was the highest at approximately 9.1 MPa.

After 56 days, the flexural strength of the control sample was approximately 9.0 MPa, and the flexural strength increased as the amount of AMFs increased.

Moreover, when the amount of AMFs was the same, the 56-day flexural strength of the sample using 0.2% CNTs was relatively greater than that of the sample using 0.1% CNTs. The 56-day flexural strength of the C2A2 LWA mortar sample using 0.2% CNTs and 20 kg/m^3^ AMFs was the highest at approximately 11.2 MPa. This value was approximately 24.4% higher than that of the control sample.

This is attributed to the crack control and crosslinking effects of CNTs and AMFs [40,41,42] . This phenomenon is likely caused by the interaction of CNTs and AMFs. Specifically, this is due to the entrainment of CNTs, which can act as a bridge across microcracks, and AMFs, which exhibit excellent crack control [40,41,42].

### 3.7. Microstructural Analysis

**Figure 11:**
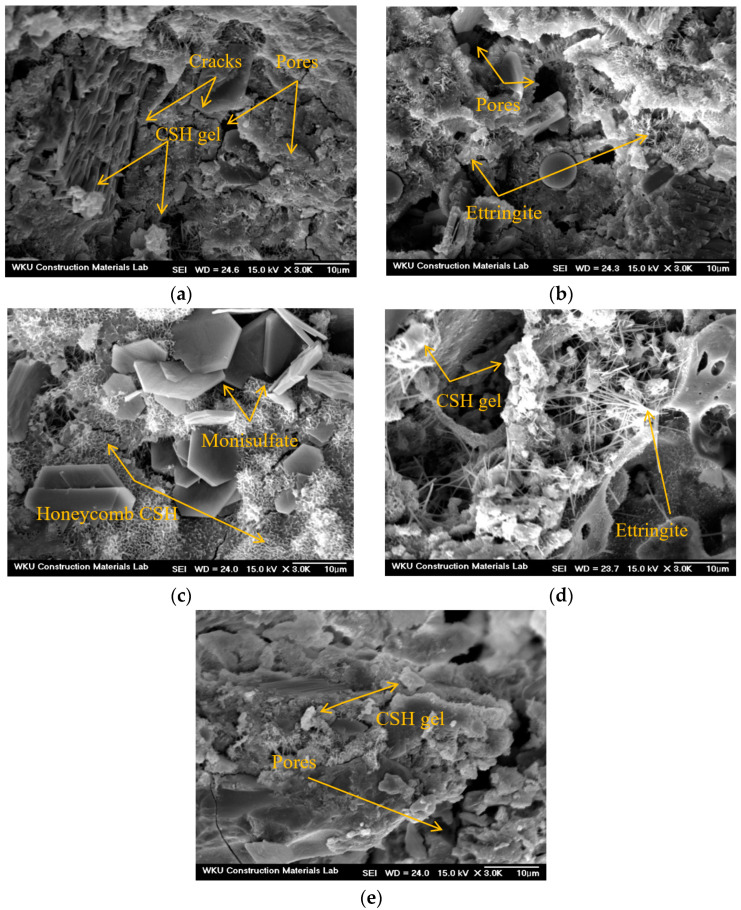
SEM images of 28-day samples (3000×): (**a**) control, (**b**) C1A0, (**c**) C1A2, (**d**) C2A0, (**e**) C2A2.

Figure 11 shows the SEM images of certain 28-day samples. Voids and microcracks were observed in the matrix of the control sample without CNTs or AMFs, along with certain hydrates. In the C1A0 sample with 0.1% CNTs, voids, ettringite, and calcium silicate hydrates (CSHs) were observed. In the C1A2 sample, a honeycomb-shaped CSH was formed around ettringite and monosulfate, and a relatively homogeneous matrix was observed compared to the control and C1A0 samples. In the C2A0 sample, pores and a somewhat less dense structure were observed, believed to have resulted from the lowest 28-day compressive strength. For the C2A2 sample with 0.2% CNTs and 20 kg/m^3^ AMFs, numerous hydrates and a dense matrix were observed.

**Figure 12:**
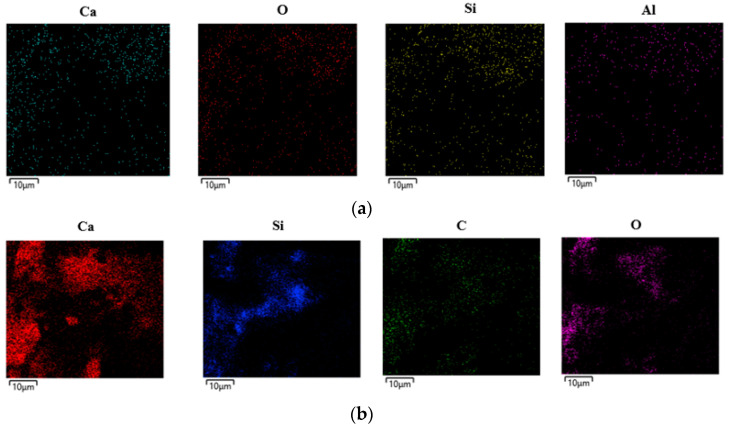
EDS mapping results of 28-day samples: (**a**) control, (**b**) C2A2.

Figure 12 and Table 6 show the EDS mapping results of the control and C2A2 samples and the EDS spot analysis results of the CSH gel within each sample, respectively. The elements detected in each sample were Ca, O, Al, Si, C, etc. In general, when the Ca/Si ratio in the CSH gel is low, the Ca^2+^ concentration is low, and strength development is delayed [43]. In addition, when the Ca/Si ratio is greater than 1.50, the stability of the gel decreases, and the CSH gel with reduced stability negatively affects the performance of the cement composite [44,45].

As is evident from Table 6, the Ca/Si ratio of the control sample was approximately 2.69, and that of the C2A2 sample, which exhibited the highest 28-day compressive strength, was approximately 1.49.

## 4. Conclusions

This study evaluated the thermal and mechanical properties of lightweight aggregate cement mortars containing CNTs and AMFs. The findings obtained can be summarized as follows:

1. As the amount of AMFs increased, the flow of the mortar sample decreased, and the flows of the C1A1 and C1A2 samples were approximately 16.6–19.6% lower than that of the C1A0 sample without AMFs.
2. In the microhydration heat test, when only the CNT was used in the LWA mortar, the time to reach the peak temperature was delayed. However, when CNTs and AMFs were used together, the peak temperature increased.
3. Through a thermal property test, the peak temperature of the sample using both CNTs and AMFs was found to be significantly higher than that of the sample using only CNTs. Thus, the peak temperature of the C1A1 and C2A2 samples using AMFs was approximately 91.5–93.8 °C, which was approximately 57.2–61.1% higher than the C1A0 sample without AMFs. In particular, the time to reach the peak temperature was approximately 15–27 min, which was 21.1–38.0% of that of the C1A0 sample.
4. The 28-day compressive strength of the control sample was approximately 35.8 MPa, and the compressive strengths of the samples using CNTs and AMFs increased as the amount of AMFs increased.
5. The 28-day split tensile strength of the sample using 20 kg/m^3^ of the AMF was approximately 3.6–3.8 MPa, which was approximately 46.1–50.0% higher than that of the sample using only CNTs. In particular, when the amount of AMFs was the same, the split tensile strength of the sample with 0.2% CNTs was greater than that of the sample with 0.1% CNTs.
6. The 56-day flexural strength of the C2A2 sample using 0.2% CNTs and 20 kg/m^3^ AMFs was the highest at approximately 11.2 MPa. This value was approximately 24.4% higher than that of the control sample.

The results of this study show that the thermal properties and mechanical performance of LWA mortar can be efficiently improved when CNTs and AMFs are used together.

In the future, additional research will be needed on the correlation between the microstructure and durability of lightweight aggregate cement composites using CNTs and AMFs.

## Figures and Tables

**Table 1:** Chemical compositions of cement samples.

	SiO_2_ (wt%)	Al_2_O_3_ (wt%)	Fe_2_O_3_ (wt%)	CaO (wt%)	MgO (wt%)	K_2_O (wt%)	Blaine (cm^2^/g)	Density (g/cm^3^)
Ordinary Portland cement	17.43	6.50	3.57	64.4	2.55	1.17	3820	3.13

**Table 2:** Physical properties of fine aggregate.

	FM	Density (g/cm^3^)	Water Absorption (%)	Unit Weight (kg/L)
Lightweight aggregate (LWA)	4.61	1.77	8.71	1010

**Table 3:** Physical properties of carbon nanotube (CNT).

	Specific Surface Area (m^2^/g)	Purity (wt%)	Bulk Density (g/mL)	Moisture Contents (wt%)
CNT	221	97.88	0.094	0.3

**Table 4:** Physical properties of amorphous metallic fiber (AMF).

	Density (g/cm^3^)	Tensile Strength (N/mm^2^)	Length (mm)
AMF	7.2	1400	15

**Table 5:** Mix proportions of cement mortars.

Mix	W/C (%)	Water (kg/m^3^)	Cement (kg/m^3^)	CNT (C*wt%)	AMF (kg/m^3^)	LWA (S*%)
Control	50	170	340	0	0	100
C1A0				0.1	0	
C1A1					10	
C1A2					20	
C2A0				0.2	0	
C2A1					10	
C2A2					20	

**Table 6:** Gel composition of control and C2A2 samples from EDS analysis.

Mix	Ca (wt%)	Si (wt%)	Al (wt%)	Fe (wt%)	K (wt%)	O (wt%)	Ca/Si (%)
Control	21.86	8.11	2.6	9.19	1.38	56.86	2.69
C2A2	38.57	25.77	2.74	-	1.46	49.33	1.49

## Data Availability

The original contributions presented in the study are included in the article; further inquiries can be directed to the corresponding author.
